# *Artemisia gmelinii* Extract Alleviates Allergic Airway Inflammation via Balancing TH1/TH2 Homeostasis and Inhibiting Mast Cell Degranulation

**DOI:** 10.3390/ijms232315377

**Published:** 2022-12-06

**Authors:** Thi Van Nguyen, Chun Hua Piao, Yan Jing Fan, Zhen Nan Yu, So-Young Lee, Chang Ho Song, Hee Soon Shin, Ok Hee Chai

**Affiliations:** 1 Department of Anatomy, Jeonbuk National University Medical School, Jeonju 54896, Republic of Korea; 2 Division of Food Functionality Research, Korea Food Research Institute, Wanju 55365, Republic of Korea; 3 Department of Food Biotechnology, University of Science and Technology, Daejeon 34113, Republic of Korea; 4 Institute for Medical Sciences, Jeonbuk National University, Jeonju 54896, Republic of Korea

**Keywords:** allergic rhinitis, asthma, TH1/TH2 balance, mast cell, *Artemisia gmelinii*

## Abstract

A new terminology “combined allergic rhinitis and asthma syndrome (CARAS)” was introduced to describe patients suffering from both allergic rhinitis (AR) and asthma. The pathogenesis of allergic airway inflammation has been well known, with the main contribution of TH1/TH2 imbalance and mast cell degranulation. *Artemisia gmelinii* has been used as an herbal medicine with its hepaprotective, anti-inflammatory, and antioxidant properties. In this study, the effect of *A. gmelinii* extracts (AGE) on the ovalbumin (OVA)-induced CARAS mouse model was investigated. AGE administration significantly alleviated the nasal rubbing and sneezing, markedly down-regulated both OVA-specific IgE, IgG_1_, and histamine levels, and up-regulated OVA-specific IgG_2a_ in serum. The altered histology of nasal and lung tissues of CARAS mice was effectively ameliorated by AGE. The AGE treatment group showed markedly increased levels of the TH1 cytokine interleukin (IL)-12 and TH1 transcription factor T-bet. In contrast, the levels of the TH2 cytokines, including IL-4, IL-5, IL-13, and the TH2 transcription factor GATA-3, were notably suppressed by AGE. Moreover, AGE effectively prevented mast cell degranulation in vitro and mast cell infiltration in lung tissues in vivo. Based on these results, we suggest that AGE could be a potential therapeutic agent in OVA-induced CARAS by virtue of its role in balancing the TH1/TH2 homeostasis and inhibiting the mast cell degranulation.

## 1. Introduction

Recently, the burden of allergic airway inflammatory disease has been reported to have risen, typically allergic rhinitis (AR) and allergic asthma (AA) [1]. Depending on their particular histology structure, the upper and lower airway allergic inflammation has typical symptoms. AR is an inflammatory disorder of the nasal mucosa, typified by symptoms of nasal itching, sneezing, anterior nasal secretions, and nasal blockage [2]. AA, on the other hand, is a chronic inflammatory pulmonary disorder characterized by reversible obstruction of the airways, causing relapses of wheezing, breathlessness, chest tightness, and cough [3]. However, both AR and AA have the same triggers; AR and AA are an abnormally exacerbated reaction towards common environmental factors, such as pollen grains or dust mites. Interestingly, AR and AA frequently coexist and interact with each other at various levels. AR is a common comorbidity of asthma that contributes to asthma severity. Moreover, AR typically precedes the development of the asthma [4]. According to epidemiology, 40% of AR patients had asthma, and 30% to 80% of asthmatic patients reported AR [5]. Therefore, a new terminology “combined allergic rhinitis and asthma syndrome (CARAS)” was introduced to describe patients suffering from both AR and AA [6].

The pathogenesis of allergic airway inflammation has been well known, with the main contribution of TH1/TH2 imbalance and mast cell degranulation [7]. More particularly, in the first time being exposed to an allergen, inhaled antigens are transported to the lymph nodes or local mucosa by lung-derived dendritic cells. Activated dendritic cells present peptides derived from the processed allergen to the naive T cells, subsequently stimulating naive T cells to differentiate into TH2 cells. TH2 cells produce interleukin (IL)-4 and IL-13, which motivate B cells to produce immunoglobulin E (IgE) into the system [8]. IgE binds to the tissue-resident mast cells (MCs) and sensitizes them. When the host is re-exposed to the allergen, sensitized MCs will be activated and release their mediator, leading to immediate allergic responses, such as rubbing, sneezing, bronchoconstriction, or increased vascular permeability [9]. MCs also contribute to the late phase of allergen-induced airway inflammation, which reflects the recruitment of innate and adaptive immune cells from the circulation and the secretion of inflammatory mediators by tissue-resident cells [10].

*Artemisia gmelinii* has been used as an additive and traditional medicine to reduce fever and relieve neonatal jaundice in traditional oriental medicine. *A*. *gmelinii* has been reported to have hepaprotective [11], anti-inflammatory [12], and antioxidant [13] properties. Previous studies showed that *A. Gmelinii* inhibited atopic dermatitis [14], suppressed inflammation from helical bacteria, protected liver injury, and liver fibrosis in mice [11]. However, the protective effect of *A. gmelinii* on allergic airway inflammatory disorders has been unknown. Therefore, the aim of this study investigates the effect of *A. gmelinii* extracts (AGE) on an OVA-induced CARAS mouse model.

## 2. Results

### 2.1. AGE Inhibited the OVA-Induced Allergic Symptoms

**Figure 1:**
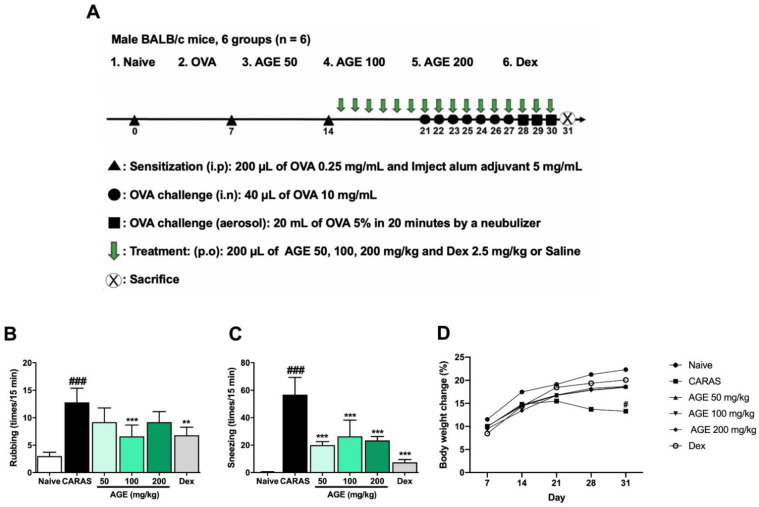
AGE inhibited the OVA-induced allergic symptoms in CARAS mice. (**A**) Animal protocol. (**B**) Rubbing. (**C**) Sneezing. (**D**) Body weight change. Mice were sensitized with OVA 3 times on days 0, 7, and 14 via intraperitoneal (i.p) injection. Mice were challenged with OVA from day 21 to day 27 by intranasal (i.n) and then from day 28 to day 30 by aerosol exposure to OVA. Mice were treated with AGE 50, 100, 200 mg/kg, or Dex 2.5 mg/kg orally (p.o) once daily from day 15 to day 30. The mice in the naive group were not sensitized, challenged, or treated. The mice in the CARAS group were treated with saline. Oral administration of AGE 50, 100, 200, or Dex 2.5 mg/kg significantly decreased the sneezing times in CARAS mice on day 27. At a dose of 100 mg/kg, AGE notably reduced the rubbing times in CARAS mice as well. All results are shown as the mean ± SD (n = 6 per group). ^#^ Compared to naive, * compared to CARAS. ^###^, *** *p* < 0.001; ** *p* < 0.01. CARAS = combined allergic rhinitis and asthma syndrome; AGE = *Artemisia gmelinii* extracts; Dex = dexamethasone; OVA = ovalbumin.

To examine the effects of AGE on clinic nasal symptoms, the number of nasal rubbings and sneezing were counted for 15 min immediately after the last nasal challenge on day 30. After exposure to OVA, mice in the CARAS group represented significant increases in nasal rubbing and sneezing compared with those in the naive group. The frequency of rubbing was notably relieved in CARAS mice that receive treatment with AGE 100 mg/kg; the number of times sneezing also was significantly decreased by the treatment with AGE 50, 100, and 200 mg/kg (Figure 1B,C). The body weight change of CARAS mice was notably decreased after the last challenge when compared with the naïve group. These other treatment groups have no difference in body weight change when compared with naïve or CARAS group. The decreased body weight of CARAS mice can be explained by the effect of the OVA challenge for the whole 2 weeks. However, the treatment of AGE or Dexa recovered it (Figure 1D).

### 2.2. AGE Treatment Decreased the Infiltration of Inflammatory Cells in NALF and BALF

**Figure 2:**
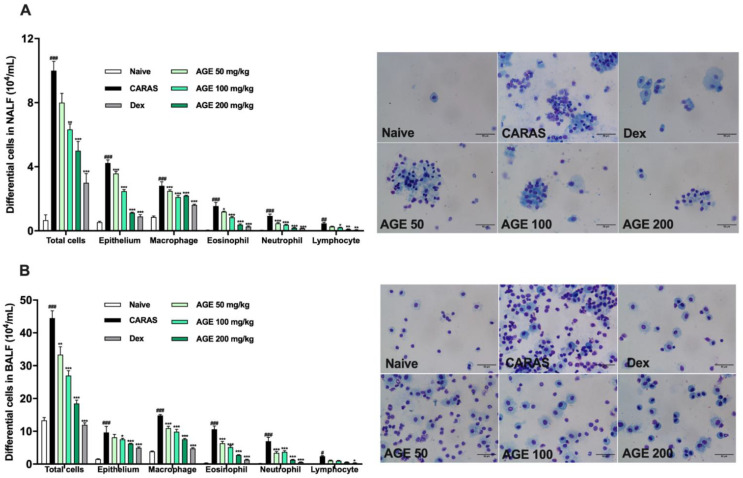
AGE suppressed the infiltration of differential inflammatory cells in both NALF and BALF of CARAS mice. (**A**) Differential inflammatory cells in NALF. (**B**) Differential inflammatory cells in BALF. The number of epithelial cells and inflammatory cells (eosinophil, neutrophil, and macrophage) were majorly increased in both NALF and BALF of the CARAS group compared with the naive group, and those cells numbers were significantly decreased by AGE 50, 100, 200 mg/kg, and Dex 2.5 mg/kg. All results are shown as the mean ± SD (n = 6 per group). ^#^ Compared to naive, * compared to CARAS. ^###^, *** *p* < 0.001; ^##^, ** *p* < 0.01, *p* < 0.05. CARAS = combined allergic rhinitis and asthma syndrome; AGE = *Artemisia gmelinii* extracts; Dex = dexamethasone; BALF = bronchoalveolar lavage fluid. Scale bar = 50 µm.

To determine whether AGE has an anti-inflammation effect on both upper and lower airways, NALF and BALF were quantified. The total cell number in NALF and BALF was determined by a microscope. In the CARAS group, total infiltrated cell numbers in NALF and BALF were significantly more abundant than in the naive group. However, at all doses of AGE 50, 100, and 200 mg/kg, the total cells in NALF and BALF were greatly decreased, as with the treatment with Dex 2.5 mg/kg (Figure 2A,B). The results of evaluating the quality of NALF and BALF showed a notable increase in the number of leukocytes, such as eosinophils, neutrophils, lymphocytes, macrophages, and epithelial cells, in the CARAS group compared with the naive group. By contrast, AGE treatment at a dose of 100 and 200 mg/kg significantly inhibited the infiltration of inflammatory cells, especially eosinophils, in both NALF and BALF compared with the CARAS group (Figure 2A,B).

### 2.3. AGE Ameliorated Nasal Mucosa Swelling, Goblet Cell Hyperplasia, and Infiltration of Eosinophil in the Nasal Tissue

**Figure 3:**
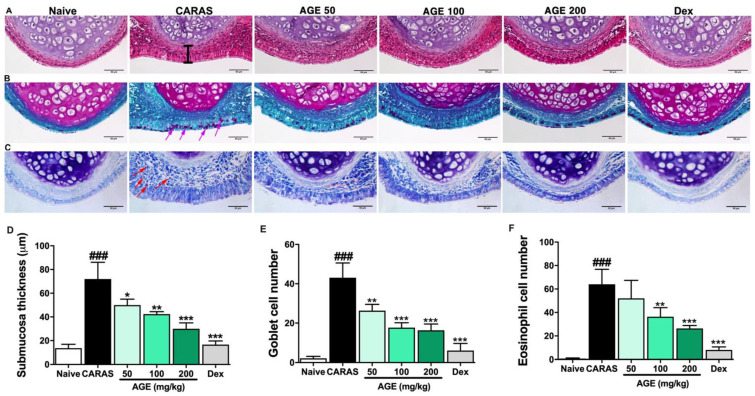
AGE ameliorated nasal mucosa swelling and goblet cell hyperplasia in CARAS mice. (**A**) H&E staining, (**B**) PAS staining. (**C**) Giemsa staining. (**D**) Submucosal thickness. (**E**) Goblet cell number in the nasal epithelial. (**F**) Eosinophil number infiltrated in the nasal tissue. All pictures were at a magnification of 400×. By H&E staining, abnormal morphology of nasal tissue was observed in the CARAS group: the sub-epithelium was significantly infiltrated by inflammatory cells, the epithelium was swelling. However, it was considerably reverted in the treated group with AGE 200 mg/kg and Dex 2.5 mg/kg. By PAS staining, the goblet cells appeared with a violet color. Goblet cell hyperplasia in the epithelial layer of the CARAS mice was considerably alleviated by AGE 50 and 100 mg/kg. The infiltration of eosinophils was detected by Giemsa staining. The majored infiltration of eosinophil in the CARAS mice was reduced by the treatment with AGE. The purple head arrows indicated goblet cells; the redhead arrows indicated eosinophils. All results are shown as the mean ± SD (n=6 per group). ^#^ Compared to naive, * compared to CARAS. ^###^, *** *p* < 0.001; ** *p* <0.01; * *p* <0.05. CARAS = combined allergic rhinitis and asthma syndrome; AGE = *Artemisia gmelinii* extracts; Dex = dexamethasone; H&E = hematoxylin and eosin; PAS = periodic acid–Schiff. Scale bar = 50 µm.

Histological alteration of the nasal mucosa was observed by H&E staining. In the CARAS group, an abnormal morphology in nasal mucosa was found. More specifically, the nasal sub-epithelium was invaded by an abundance of inflammatory cells, and the epithelial layer in the nasal septum was swelled. It leads to a considerable thickened nasal septum in the CARAS mice compared with the naive group. The treatment of AGE 200 mg/kg and the Dex 2.5 mg/kg group notably ameliorated both the accumulation of inflammatory cells and the swelling of the epithelial layer (Figure 3A,D). Rhinorrhea is a common nasal allergic symptom in AR [15]. Major secretions come from goblet cells in the nasal epithelial layer. In this study, by PAS stain, the goblet cell hyperplasia was observed in the CARAS group but it was alleviated by oral treatment of AGE 50, 100, and 200 mg/kg (Figure 3B,E). The goblet cell appeared in violet color in the epithelial layer. A majored penetration of eosinophils into the sub-mucosal tissue of CARAS mice was observed by Giemsa staining. However, it was obviously reduced by the treatment with AGE or Dex (Figure 3C,F).

### 2.4. AGE Effectively Suppressed Inflammation, Mucus Secretion, and Collagen Deposition in the Lung Tissue

**Figure 4:**
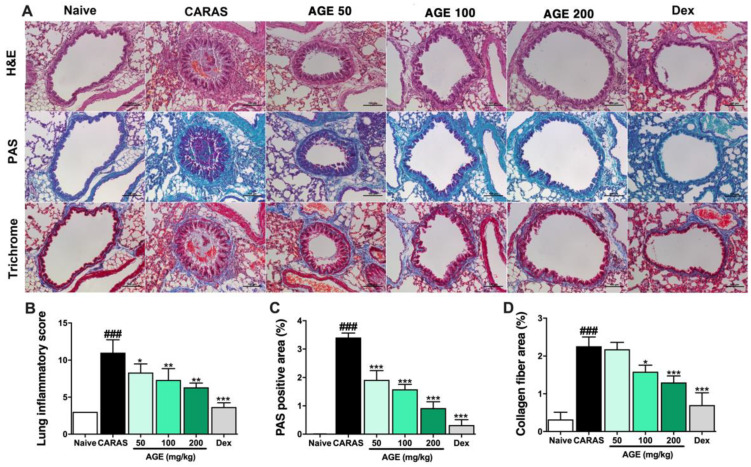
AGE effectively suppressed inflammation, mucus secretion, and collagen deposition in the lung tissue of CARAS mice. (**A**) The histology of lung tissue. (**B**) Lung inflammation score. (**C**) PAS-positive area. (**D**) Collagen fiber area. All pictured were at a magnification of 200×. By H&E staining, typical inflammation features were observed in lung tissue of CARAS mice: bronchial smooth muscle thickened, bronchial epithelium cells were swelling, and alveoli surrounding bronchia were majorly penetrated with inflammatory cells. By PAS staining, CARAS mice showed an increase in goblet cells resulting in over-secreted mucus into the lumen of the bronchia. By trichrome staining, the collagen fiber (which was stained by blue color) was abundantly expressed surrounding the bronchi and vessels in the lung tissue of CARAS mice. However, the inflammation and goblet cell hyperplasia in CARAS mice were considerably attenuated by AGE and the collagen fiber deposition also lessened in the treatment group with AGE. All results are shown as the mean ± SD (n = 6 per group). ^#^ Compared to naive, * compared to CARAS. ^###^, *** *p* < 0.001; ** *p* < 0.01; * *p* < 0.05. CARAS = combined allergic rhinitis and asthma syndrome; AGE = *Artemisia gmelinii* extracts; Dex = dexamethasone; H&E = hematoxylin and eosin; PAS = periodic acid–Schiff. Scale bars = 100 µm.

In the CARAS group, by H&E staining, the bronchus showed an irreversible airway obstruction compared with the naive group. More particularly, the bronchial epithelial showed edema and was thickened, and the alveolar septal regions surrounding bronchia were penetrated with inflammation cells. Lung inflammation score was estimated following several criteria of edema, hemorrhage, cell infiltration, the thickness of alveoli septum, and alveolar distortion [16]. By PAS stain, the goblet cell hyperplasia leads to a mucus over-secreted situation in the bronchial lumen in the CARAS group (Figure 4A,C). In contrast, analyzed lung tissue results of mice in the group AGE 100, 200, and Dex 2.5 displayed a recovery from inflammation, recovered to normal structure with thin bronchial epithelial, less infiltration of leukocytes, and the hyperplasia in lung tissue was alleviated (Figure 4A–C). Masson trichrome was performed to observe collagen fiber deposition in the lung tissue. The collagen fibers (stained by blue color) were abundantly deposited surrounding the bronchia of CARAS mice compared with naive mice. In addition, the collagen amount in AGE 200 mg/kg and Dex 2.5 mg/kg was considerably decreased compared with the CARAS group (Figure 4A,D).

### 2.5. AGE Treatment Suppressed Allergic Responses by Regulating Serum Antigen-Specific-Immunoglobulins

**Figure 5:**
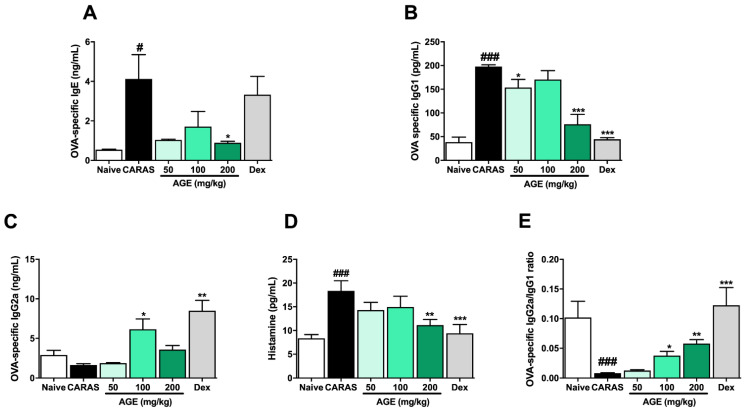
AGE suppressed allergic responses by regulating OVA-specific Igs in the serum of CARAS mice. (**A**) OVA-specific IgE, (**B**) OVA-specific IgG_2a_, (**C**) OVA-specific IgG_1_, (**D**) OVA-specific IgG_2a_/IgG_1_, and (**E**) histamine. Oral administration of AGE 200 mg/kg significantly down-regulated the levels of OVA-specific IgE, IgG1, and histamine. At the dose of 100 mg/kg, AGE also strongly up-regulated the level of OVA-specific IgG2a in the serum of CARAS mice. All results are shown as the mean ± SD (n = 6 per group). ^#^ Compared to naive, * compared to CARAS. ^###^, *** *p* < 0.001; ** *p* < 0.01, ^#^, * *p* < 0.05. CARAS = combined allergic rhinitis and asthma syndrome; AGE = *Artemisia gmelinii* extracts; Dex = dexamethasone; Ig = immunoglobulins; OVA = ovalbumin.

To investigate the effect of AGE on systemic allergic responses, the levels of OVA-specific IgE, IgG_1_, IgG_2a_, and histamine in the serum of mice were detected by ELISA. In the present study, the results showed a sharply increased level of OVA-specific IgE, OVA-specific IgG_1_, and histamine in the serum of CARAS mice compared with the naive group. In addition, those levels were remarkably down-regulated in CARAS mice treated with AGE 200 mg/kg (Figure 5A,B,D). The level of OVA-specific IgG_2a_ was notably up-regulated by AGE 100 mg/kg compared with CARAS mice (Figure 5C). The level of OVA-specific IgE in CARAS mice was remarkably down-regulated by the treatment with AGE in a dose-dependent manner but not the level of OVA-specific IgG1. However, the ratio of OVA-specific IgG2a/OVA-specific IgG1 was significantly increased by AGE dose-dependently (Figure 5E). IgG2a is a marker for Th1 cells, while IgG1 is a marker for Th2 cells. Therefore, the Th1/Th2 balance was improved by the treatment with AGE.

### 2.6. AGE Increased TH1 Cytokines and TH1 Transcription Factor in NALF and BALF

**Figure 6:**
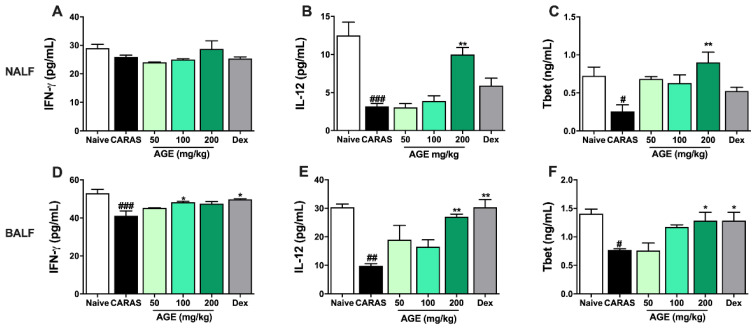
AGE increased TH1 cytokines and TH1 transcription factor in NALF and BALF. The levels of (**A**) IFN-γ, (**B**) IL-12, and (**C**) T-bet in NALF. The levels of (**D**) IFN-γ, (**E**) IL-12, and (**F**) T-bet in BALF. The levels of IL-12 and T-bet in NALF and the levels of IFN-γ, IL-12, and T-bet in BALF were significantly improved by AGE. All results are shown as the mean ± SD (n = 6 per group). ^#^ Compared to naive, * compared to CARAS. ^###^ *p* < 0.001; ^##^, ** *p* < 0.01; ^#^, * *p* < 0.05. CARAS = combined allergic rhinitis and asthma syndrome; AGE = *Artemisia gmelinii* extracts; Dex = dexamethasone; NALF = nasal lavage fluid; BALF = bronchoalveolar lavage fluid; IL = interleukin.

TH1 cytokines, such as IFN-γ, IL-12, and TH1 transcription factor T-bet, in both NALF (Figure 6A–C) and BALF (Figure 6D–F) were quantified by ELISA. The level of IL-12 and T-bet in NALF and BALF were significantly decreased in the CARAS group compared with those in the naive group. The level of IL-12 was greatly improved by AGE 200 mg/kg. The level of T-bet was markedly enhanced by the administration of AGE 50, 100, 200 mg/kg, and Dex 2.5 mg/kg. However, there is no significant difference between groups in the level of IFN-γ in NALF. The level of IFN-γ in BALF in the CARAS group was considerably decreased compared with the naive group, while there was no statistical difference between the treated group and CARAS group.

### 2.7. AGE Decrease TH2 Cytokines and TH2 Transcription Factor in NALF and BALF

**Figure 7:**
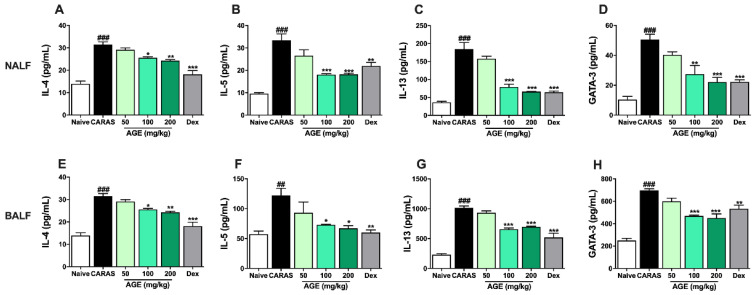
AGE decreased TH2 cytokines and TH2 transcription factor in NALF and BALF of CARAS mice. The levels of (**A**) IL-4, (**B**) IL-5, (**C**) IL-13, and (**D**) GATA-3 in NALF and (**E**) IL-4, (**F**) IL-5, (**G**) IL-13, and (**H**) GATA-3 in BALF. The levels of TH2 cytokines IL-4, IL-5, IL-13, and TH2 transcription factor GATA-3 in NALF were significantly suppressed by AGE 200 mg/kg or Dex 2.5 mg/kg. The levels of IL-4 and IL-5 in BALF also notably decreased in the AGE 200 and Dex group. All results are shown as the mean ± SD (n = 6 per group). ^#^ Compared to naive, * compared to CARAS. ^###^, *** *p* < 0.001; ^##^, ** *p* < 0.01; * *p* < 0.05. CARAS = combined allergic rhinitis and asthma syndrome; AGE = *Artemisia gmelinii* extracts; Dex = dexamethasone; NALF = nasal lavage fluid; BALF = bronchoalveolar lavage fluid; IL = interleukin.

To investigate the impact of AGE on the modulation of T helper homeostasis, TH2 cytokines (IL-4, IL-5, and IL-13) and TH2 transcription factor (GATA-3) were estimated by ELISA assay. In NALF, the levels of IL-4, IL-5, IL-13, and GATA-3 were sharply increased in the CARAS group compared with the naive group. The level of IL-4 and GATA-3 was significantly alleviated by AGE 200 mg/kg and Dex 2.5 mg/kg, while IL-5 and IL-13 were notably decreased by AGE 100 and 200 mg/kg similarly with Dex 2.5 mg/kg (Figure 7A–D). 

Similar to NALF, the levels of IL-4, IL-5, IL-13, and GATA-3 in BALF were majorly raised in the CARAS group compared with the naive group. The level of IL-4 was greatly declined by the treatment of both AGE 100 and 200 mg/kg, while the level of IL-5 was markedly reduced in the administration of AGE 200 mg/kg. There was a downtrend in the levels of IL-13 and GATA-3 in the mice treated with AGE and Dex compared with CARAS mice (Figure 7E–H). 

### 2.8. AGE Decreased the Expression of Transcription Factors in Lung Tissues of CARAS Mice

**Figure 8:**
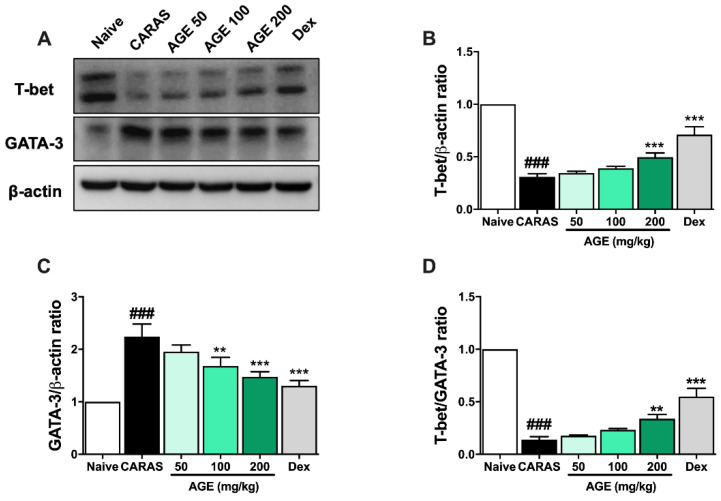
AGE decreased the expression of transcription factors in the lung tissues of CARAS mice. (**A**) Western blot data. (**B**) The relative level of T-bet and (**C**) GATA-3 expression in lung tissue; (**D**) T-bet/GATA-3 ratio. The expression of T-bet in the lung tissue of CARAS mice was notably increased by the treatment with AGE 200 mg/kg or Dex. In contrast, the expression of GATA-3 was significantly suppressed by the treatment of AGE or Dex. Moreover, the T-bet/GATA-3 ratio, which represents the Th1/Th2 balance, was remarkably improved by the treatment of AGE 200 mg/kg or Dex. All results are shown as the mean ± SD (n = 6 per group). ^#^ Compared to naive, * compared to CA-RAS. ^###^, *** *p* < 0.001; ** *p* < 0.01. CARAS = combined allergic rhinitis and asthma syn-drome; AGE = Artemisia gmelinii extracts; Dex = dexamethasone; GATA-3 = GATA binding protein 3; T-bet = T-box expressed in T cells.

To evaluate the TH1-transcription factor T-bet and TH2-transcription factor GATA-3 expression in the lung tissue of mice, the Western blot assay was performed. The expression of T-bet was majorly lessened in the CARAS group when compared with the naïve group. The low level of T-bet expression in the lung tissue of CARAS mice was notably reversed by the treatment with AGE 200 mg/kg or Dex (Figure 8A,B). In contrast, the expression of GATA-3 in the CARAS group was seriously up-regulated when compared with the naïve group and it was significantly suppressed by the treatment of AGE or Dex (Figure 8A,C). Moreover, the T-bet/GATA-3 ratio, which represents the Th1/Th2 balance, was remarkably improved by the treatment of AGE 200 mg/kg or Dex (Figure 8D).

### 2.9. AGE Directly Inhibited Mast Cell Degranulation In Vitro and Mast Cell Infiltration in the Nasal and Lung Tissue In Vivo Experiment

**Figure 9:**
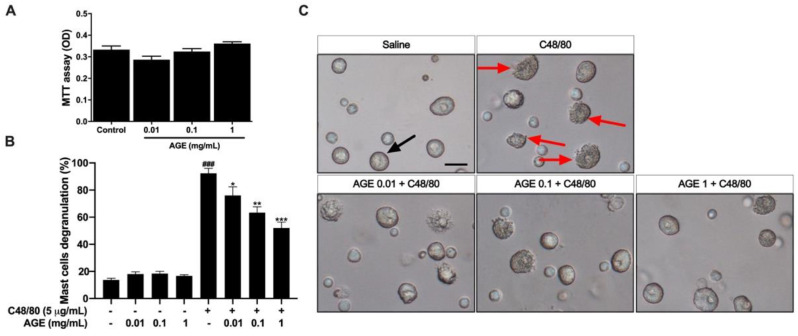
AGE strongly inhibited C48/80-induced mast cell degranulation. (**A**) MTT assay, (**B**) RPMC degranulation ratio, and (**C**) morphology of RPMC under microscopy. RPMCs were pretreated with AGE (0.01, 0.1, 1 mg/mL) or saline for 10 min at 37 °C and then incubated with MTT reagent for 1.5 h (in MTT assay) or incubated with C48/80 (5 µg/mL) or saline for 15 min (in RPMC degranulation experiment). AGE not only significantly alleviated the C48/80-induced RPMC degranulation, but also was not toxic to RPMCs. Each group was performed in duplicate. All results are shown as the mean ± SD. ^#^ Compared to naive, * compared to CARAS. ^###^, *** *p* < 0.001; ** *p* < 0.01; * *p* < 0.05. The black arrow indicates normal mast cell; the red arrow indicates degranulated mast cell. CARAS = combined allergic rhinitis and asthma syndrome; AGE = *Artemisia gmelinii* extracts; Dex = dexamethasone; C48/80 = compound 48/80. RPMC = rat peritoneal mast cell. Scale bar = 10 µm.

An RPMC experiment was performed to investigate whether AGE could affect mast cell degranulation. In the absence of C48/80, a mast cell degranulation reagent, AGE did not significantly raise the mast cell degranulation ratio. Moreover, by MTT assay, AGE has no toxicity on the mast cell (Figure 8A). Without pretreating with AGE, C48/80 sharply increased the mast cell degranulation ratio compared with the control group. Interestingly, by pretreating with AGE 0.1 and 1 mg/mL, the mast cell was protected from C48/80 considerably (Figure 9B,C).

**Figure 10:**
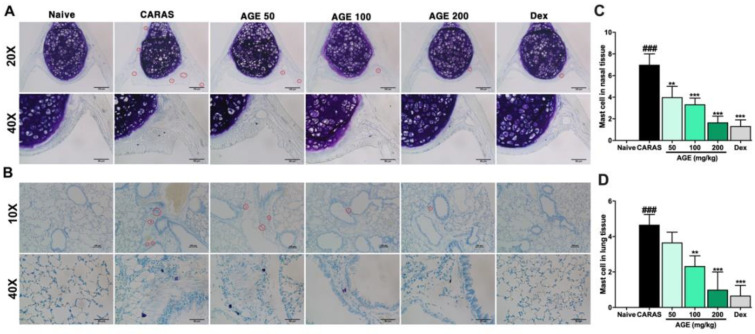
AGE strongly inhibited mast cell infiltration in the (**A**) nasal and (**B**) lung tissues of CARAS mice. The number of mast cells in the (**C**) nasal and (**D**) lung tissues. All pictures were at a magnification of 100×. By Toluidine blue, mast cells appeared in dark blue dots. The mast cell infiltration of the lung tissues in CARAS mice was notably decreased by the treatment with AGE 100, 200 mg/kg, or Dex 2.5 mg/kg. The red circles indicate mast cells. ^#^ Compared to naive, * compared to CARAS. ^###^, *** *p* < 0.001; ** *p* < 0.01. CARAS = combined allergic rhinitis and asthma syndrome; AGE = *Artemisia gmelinii* extracts; Dex = dexamethasone. Scale bars = 100 µm.

By toluidine blue staining, the mast cell was stained as a dark blue dot. The lung tissue and nasal tissue of the CARAS group were markedly infiltrated by mast cells compared with the naive group. However, the mast cell infiltration in the nasal and lung tissue was considerably suppressed by AGE or Dex treatment (Figure 10A,B).

## 3. Discussion

Much evidence has been reported that AR and asthma are closely associated and a bidirectional link exists between them [17]. AR is a major risk factor to develop asthma and the presence of asthma negatively impacts the nasal symptoms and severity of the AR [4]. AR and asthma are both chronic inflammation disorders with similar pathophysiology, with different symptoms due to their physical structure. CARAS was described as a single disease with two manifestations of both AR and asthma [6]. In this study, we established an OVA-induced CARAS mouse model by three times sensitization with OVA followed by three times aerosol OVA challenge, then seven times daily intranasal OVA challenge. The male mice were used in this study because estrous cycles might cause data variability. The nose hypersensitivity in CARAS mice was observed with regularly appearing nasal symptoms, such as rubbing and sneezing. The nasal septum was infiltrated by various inflammatory cells, and goblet cell hyperplasia was observed in the epithelial layer. In the lower airway, a characterized asthma lung histology was exhibited with epithelial edema, blobs of mucus present in the airway, and smooth muscle in the bronchi was thickened by increasing collagen fiber deposition. In addition, the lung tissue was invaded by a number of inflammatory cells. The innate immune system was activated, leading to a rising number of eosinophils in both NALF and BALF. The level of IgE also was up-regulated, and the T helper cell differentiated toward to TH2 cell, causing the imbalance between TH1 and TH2 cells. The level of IL-4, IL-5, IL-13, and TH2 transcription factor GATA-3 increased, while TH1 cytokine IL-12 and TH1 transcription factor T-bet levels were decreased in both NALF and BALF. That demonstrated that the CARAS mice were successfully established with both inflammation features in the upper and lower airway tract.

The sensitization with allergen and aluminum can stimulate the immune system to produce antibody IgE, which, subsequently, lodges in mast cells to form the complex IgE-primed mast cell [18]. With repeated exposure to an allergen, more IgE is produced and the tissue-resident mast cells are activated, degranulated, and release their mediator, which stimulates sensory nerves, causing increased nasal sensations, such as itching, rubbing, and sneezing [19]. In this study, the nasal symptoms in CARAS mice treated with AGE were considerably decreased compared with positive control mice. Therefore, we checked the level of OVA-specific IgE and histamine to demonstrate the anti-allergic effect of AGE. Interestingly, the levels of both IgE and histamine were significantly down-regulated in CARAS mice treated with AGE. IgG_2a_, which is a marker of the TH1 cell [20], was greatly enhanced by the treatment with AGE. This implies that AGE has an anti-allergic effect in the OVA-induced CARAS mouse model.

In the later phase, epithelial mast-cell-released mediators create the gap between epithelial cells, leading to allergen entering lamina propria and activating the mast cells here [21]. Eosinophils are the main source of TGF-β in bronchial, which is involved in tissue remodeling via fibroblast proliferation and increased production of collagen and glycosaminoglycans [22]. Once activated, eosinophils release toxic products, which are able to damage airway epithelial cells, epithelial cell swelling, or shedding [23]. To evaluate the effect of AGE on the later phase of allergic inflammation, the NALF and BALF were analyzed; the histological changes in nasal tissue and lung tissue were evaluated. The total cell number in both NALF and BALF was notably decreased in the mice treated with AGE. Among the infiltrated cells in the NALF and BALF, the presentation of eosinophil was remarkedly declined by AGE. The damage to the epithelial layer caused epithelial shedding in both upper and lower airways, which was verified by the majorly increasing epithelial cell number in both NALF and BALF of the CARAS group. However, the shedding of epithelial cells was ameliorated by the treatment with AGE. According to nasal tissue histological examination, the inflammation features in nasal subepithelial and lung tissue of CARAS mice were clearly alleviated by AGE. The edematous nasal and bronchial epithelial layers were considerably attenuated by AGE. In addition, by trichrome staining, AGE showed the inhibition of collagen fiber syncretization ability in the lung tissue of CARAS mice. Further, the mucus secretions in both nasal and lung tissue were suppressed by treatment with AGE. From these results, it is reasonable to hypothesize that AGE has a potent anti-inflammatory effect in both upper and lower airway systems.

Adaptive immune responses are involved in the development of allergic diseases, such as bronchial asthma and AR [24]. Sensitization to an allergen elicits a TH2 cell response to induce B cells to switch to IgE production by releasing IL-4 and IL-13 [10]. TH2-derived IL-5 is the main cytokine that activates eosinophil in the late-phase response to antigen challenge [25]. A specific inhibitor of interleukin-5 could attenuate pulmonary inflammation [26]. Otherwise, IL-13 is primarily produced by TH2 cells [27] and causes mucus hypersecretion by the replacement of epithelial cells with goblet cells [28]. IL-13 is homologous to IL-4 and, together with IL-4, acts on mononuclear phagocytic cells, endothelial cells, epithelial cells, and B cells [29]. Thus, IL-13 also can promote B cells to produce IgE and contribute to the development of allergies. GATA-3, which is known as the TH2 transcription factor, regulates TH2 cytokine expression by directly binding to the promoters of the IL-5 and IL-13 genes, and is also involved in the remodeling of the chromatin structure to open the IL-4 locus [30]. In this study, the administration of AGE suppressed the TH2 cytokines IL-4, IL-5, and IL-13 in the CARAS mice. It is in agreement with the decrease in the level of OVA-specific IgE and the down infiltration of eosinophil and goblet cells in the airway epithelial in the AGE-treated mice. GATA-3 has been well known as a master regulator of the TH2 cell differentiation [31]. GATA-3 can also repress TH1 cytokine production by antagonism with IL-12 [32]. However, T-bet, which is a regulator of TH1 cell, binds to and promotes repressive chromatin modifications at the GATA-3 locus and inhibits the expression of GATA-3 in TH1 cells [33]. Moreover, the deficiency of T-bet in the mice resulted in airway eosinophilia, overexpression of TH2 cytokines, and airway hyper-responsiveness [34]. In this study, the level of T-bet was significantly enhanced and the level of GATA-3 was notably inhibited by the oral treatment of AGE. From these results, AGE could have a positive effect on the adaptive immune system in the CARAS mouse model by enhancing the TH1/TH2 balance.

Mast cells have been well known to be the main effector cells in the pathogenesis of both allergic response and inflammatory processes [35]. The mast cell degranulation is the first event that starts the allergic reaction chain. Together with TH2 cells, mast cells accumulate inflammation cells, especially eosinophils, in large numbers and activate them [36]. In this study, the high infiltration of mast cell numbers in the lung tissue of CARAS mice was alleviated by AGE or Dex. Moreover, to investigate the impact of AGE on mast cell in vitro, the RPMC degranulation and MTT assay were performed. The results demonstrated that AGE was not toxic to RPMCs and markedly attenuated the degranulation ratio of RPMCs when incubated with C48/80. This study provides evidence that AGE has a strong effect on the prevention of mast cell degranulation and that it may suppress allergic inflammation. The significant decrease in histamine levels in AGE-treated mice was correlated to these results, suggesting that AGE could alleviate allergic inflammatory symptoms via inhibiting mast cell degranulation in the allergic immune response.

## 4. Materials and Methods

### 4.1. Preparation of Artemisia gmelinii Extract

AGE used in this study was provided by Natural F&P Co. Ltd. (Cheongju-si, Republic of Korea). Briefly, *Artemisia gmelinii* herbs were collected from Jecheon-si (Chungcheong-do, Republic of Korea) and Youngcheon (Gangwon, Republic of Korea). Raw *A. gmelinii* was dried, powdered, and then extracted with 15 times volumes of 50% ethanol by heating (±50 °C) for 6 h. AGE was subsequently filtered using a cartridge filter. Using a Rotavapor R-210 (BÜCHI Labortechnik AG, Flawil, Switzerland), the filtered extract was concentrated under a vacuum at 50 °C and then dried. The AGE was standardized by scopolin, a component of AGE. The AGE was dissolved in saline prior to use in the experiments.

### 4.2. CARAS Model Establishment and Treatment

Male six-week-old BALB/c mice and twelve-week-old rats were obtained from Damool Science (Dae-jeon, Republic of Korea). The mice and rats were housed under laboratory conditions for 1 week before experiments, with an average temperature of 23 ± 3 °C, the humidity of 50 ± 10%, and 12 h light/dark cycles. All experimental procedures were performed following the guidelines of the Institutional Animal Care and Use Committee of the Jeonbuk National University Medical School (JBNU 2021-0115) and were approved by the National Institutes of Health.

BALB/c mice were randomly separated into 6 groups (n = 6): (1) naive group, (2) CARAS group (OVA + saline), (3) AGE 50 group (OVA + AGE 50 mg dry weight/kg), (4) AGE 100 group (OVA + AGE 100 mg dry weight/kg, (5) AGE 200 group (OVA + AGE 200 mg dry weight/kg), and (6) dexamethasone (Dex) group (OVA + Dex 2.5 mg dry weight/kg). Mice were sensitized by intraperitoneal injection 3 times on day 0, 7, and 14 with 200 µL saline suspension including 50 μg OVA (Grade V, Sigma, St. Louis, MO, USA) and 25 µL of aluminum hydroxide (Imject Alum Adjuvant, Thermo Scientific, Rockford, MD, USA). Mice were treated orally with various doses of AGE (50, 100, or 200 mg/kg) or Dex (2.5 mg/kg) daily from day 15 to day 30. Mice in the CARAS group were treated with saline. After treatment 1 h, mice were challenged intranasally with 20 µL of 10 mg/mL OVA in each nasal cavity from day 21 to day 27, then by aerosol exposure to OVA (5%, *w/v*, in saline) for 20 min using an ultrasonic nebulizer (NE-U17, Omron Co., Tokyo, Japan) from day 28 to day 30. Mice were sacrificed after the last OVA challenge 24 h (Figure 1A).

### 4.3. Nasal Symptoms

After the last OVA intranasal challenge on day 30, the mice’s behavior was recorded by a camera immediately for 15 min. Rubbing and sneezing time were counted (blind check) by another partner.

### 4.4. Nasal Lavage Fluid (NALF), Bronchoalveolar Lavage Fluid (BALF) Collection, and Analysis

NALF and BALF were collected as described previously [37]. After sacrifice, 1 mL sterile saline was pumped through the trachea into the nasal cavities by a catheter. NALF was collected from the anterior naris. Next, BALF was taken by perfusing 1 mL sterile through the opened trachea position toward the lung and withdrawn through the cannula. NALF and BALF were centrifuged at 10,000 rpm for 10 min at 4 °C. The supernatant was placed into another Eppendorf tube and stored at −80 °C for further analysis. The cell pellet was resuspended in saline. A total of 150 µL of NALF and BALF was centrifuged onto slides by the cytospin device (Centrifuge 5403, Eppendorf, Hamburg, Germany) at 1000 rpm for 10 min. The total number of cells was counted using a hemocytometer. Differentiation of cells in the NALF and BALF to eosinophils, neutrophils, macrophages, lymphocytes, and epithelial cells was determined by the Diff-Quik Staining kit (1-5-1 Wakinohama-Kaigandori, Chuo-Ku, Kobe, Japan). The inflammatory cells were counted at 400× magnification under a light microscope (Leica, Teaneck, NJ, USA)

### 4.5. Determination of OVA-Specific Immunoglobulins and Histamine in Serum

Blood samples from each mouse were collected at the time of sacrifice, then immediately centrifuged for 10 min at 10,000 rpm, 4 °C, to isolate serum. The serum was stored at −80 °C for further analysis. ELISA was performed on serum samples to detect OVA-specific IgE (439807; BioLegend, Inc., San Diego, CA, USA); OVA-specific IgG2a (3015; Chondrex, Inc., Washington, DC, USA); OVA-specific IgG1 (500830, Cayman, MI, USA); and histamin (ab 213975, Abcam, Cambridge, UK) level according to the manufacturer’s instructions. Absorbance was measured using the Bio-Rad 680 microplate reader (Bio-Rad Laboratories, Inc., Hercules, CA, USA) at 450 nm.

### 4.6. Histological Examination

Head and lung tissue were processed as described in previous studies [37]. For histopathology examination, the head and lung tissues were fixed with 10% neutral buffered formalin for 3 days at 23~25 °C. Then, the samples were dehydrated with a gradually increasing concentration series of ethanol, the ethanol cleared by xylene, and then embedded in paraffin. The head tissues were decalcified in a Calci Clear-Rapid (National Diagnostics) solution for 2 days at room temperature (20~25 °C) before the dehydrating process. The tissues were sectioned at 4.5 µm thickness for head and lung tissue. Hematoxylin and eosin (H&E) (Sigma, St. Louis, MO, USA), Toluidine blue (Sigma, St. Louis, MO, USA), periodic acid–Schiff (PAS) (Sigma, St. Louis, MO, USA), and Trichrome (Sigma, St. Louis, MO, USA) staining was performed to estimate general morphology and observe mast cell infiltration and goblet cell hyperplasia in nasal and lung tissues. The membrane thickness of the nasal sub-mucosa was measured by NIS-Elements BR 4.5 software. The eosinophil counting, PAS, and the collagen fiber deposition-positive area in the nasal or lung tissue were measured by the software Fiji with a total of 1 section of 3 mice in each group.

### 4.7. Quantification of Cytokines

The supernatant was collected from the NALF and BALF for quantitating cytokine release by ELISA kit following the manufacturer’s instructions. Cytokine quantitation kits were used to measure cytokine levels, including those of IL-4, IL-5, IL-13, IL-12, IFN-y (R&D Systems, Inc., Minneapolis, MN, USA), T-bet (LSBio, Washington, DC, USA), and GATA-3 (Finetest, Wuhan, China). All assays were performed in duplicate, using standard solutions; NALF and BALF samples were transferred to a 96-well plate, which was prior coated with a target cytokine capture antibody, incubated for 2 h at room temperature, then washed 4 times. After being incubated 2 h with an appropriate biotin-conjugated antibody, the wells were aspirated and washed again 4 times; then, a horseradish peroxidase was added to each well. Removing the excess HRP conjugate by washing, a substrate solution was added to convert the solution to a detectable form. Stop solution was added to each well and the optical density of each well read at 450 nm immediately.

### 4.8. Western Blot

The presence of TH1-transcription factor T-bet and TH2-transcription factor GATA-3 were determined by Western blot. Lung tissues were homogenized with RIPA buffer (EBR001-1000, Enzynomic, Daejeon, Republic of Korea) to extract proteins, then quantified by BSA standard with Bradford dye (Bio-Rad Laboratories, Inc., Hercules, CA, USA). The samples were loaded onto an SDS-PAGE 10% and then transferred onto an activated polyvinylidene difluoride (PVDF) membrane (Bio-Rad Laboratories, Inc., USA). The membrane was blocked with 5% skim milk for approximately 1 h, then incubated with diluted primary antibody at 1/1000 BSA 5% overnight at 4 °C by an orbital shaker. After washing off the primary antibody by TBST, the membrane was then incubated with a secondary antibody for about 1 h at room temperature. The blot was detected by an ECL substrate. The density of bands from the membrane was measured using ImageJ software.

### 4.9. Rat Peritoneal Mast Cell Degranulation

Rat peritoneal mast cells (RPMCs) were isolated as described previously [38]. In brief, rats were anesthetized with ether and injected with 10 mL of phosphate-buffered saline into the peritoneal cavity, and the abdomen was gently massaged for about two min. The peritoneal cavity was opened, an additional 50 mL phosphate-buffered saline was added, then they continued to be massaged (avoid touching the liver). The fluid was aspirated to another 50 mL tube using a Pasteur pipette, and then centrifuged for 10 min at 1000 rpm, 4 °C. The supernatant was discarded and RPMCs resuspended with HEPES (1 × 10^6^ cells/mL). Two hundred microliter of RPMCs were pretreated with 25 µL saline (control group) or AGE (0.01, 0.1, 1 mg/mL) for 10 min at 37 °C, and then incubated with 25 µL compound 48/80 (C48/80) or saline for 15 min. After incubation, the mast cell degranulation rate (the degranulated mast cells/total mast cells × 100) was counted under a microscope.

### 4.10. Mast Cell Viability Assay

To test the toxicity of AGE on mast cells, the MTT assay (Abcam, Cambridge, UK) was performed according to the manufacturer’s instructions. A total of 100 µL RPMCs (1 × 10^6^ cells/mL) was added in a clear 96-well plate free-coated. RPMCs were incubated with various concentrations of AGE (0.01, 0.1, 1 mg/mL) at 37 °C for 15 min. Next, RPMCs were incubated with MTT reagent for one and a half hours at 37 °C. After incubation, the MTT reagent–supplement media was removed. A total of 150 µL of MTT solvent was added into each well. The plate was shaken for 15 min (avoiding light) and absorbance read at OD 590 nm.

### 4.11. Statistical Analysis

The experimental data were analyzed using GraphPad Prism 6.0 software (v5.0, La Jolla, CA, USA). Differences between groups were analyzed using one-way ANOVA, followed by Turkey’s test or two-way ANOVA, followed by Bonferroni’s multiple comparison. The data are presented as means ± standard error (SE) of independent experiments. *p* < 0.05 was considered to indicate the significance of the data.

## 5. Conclusions

In conclusion, it has been shown that AGE treatment may alleviate both upper and lower airway allergic inflammation by suppressing TH2 cytokines and enhancing TH1 cytokines, balancing the TH1/TH2 ratio. Moreover, AGE strongly inhibits mast cell degranulation and infiltration into lung tissue, which attenuates the allergic response. These obtained results suggested that AGE treatment could be a promising candidate for the prevention and treatment of allergic airway diseases.

## Data Availability

The data presented in this study are available on request from the corresponding author.
